# Prevaccination screening of health care workers for immunity to measles, rubella, mumps and varicella in a developing country. What do we save?

**DOI:** 10.1186/1753-6561-5-S6-P277

**Published:** 2011-06-29

**Authors:** E Alp, F Cevahir, S Gokahmetoglu, H Demiraslan, M Doganay

**Affiliations:** 1 Erciyes University, Kayseri, Turkey

## Introduction / objectives

Immunity to measles, rubella, mumps, and varicella (MMRV) is an important part of infection control among healthcare-workers (HCWs).To evaluate the cost effectiveness of prevaccination screening of HCWs against MMRV.

## Methods

A structured survey form,including data of age, gender, job category, length of employment, history of MMRV infections and status of MMRV vaccinations, was applied to HCWs.Serologic screening for MMRV was performed on HCWs using enzyme-linked immunosorbent assay (EIA).The cost of each test for MMR was €2.5 and €5 for varicella.In a cost-effectiveness analysis, MMR^®^(supplied by Ministry of Health from Serum Institute of India for €2.5) and Varilrix^®^(GlaxoSmithKline-cheapest vaccination in the marketplace-€25) vaccines were used.

## Results

One thousand and two hundred fifty-five HCWs were tested.Of these, 798 (64%) were female and the age ranged from 19 to 60 years (median 30).The median length of employment was 5 (≤1-47 years).Of the employees examined, 94% were immune to measles, 97% to rubella, 90% to mumps, 98% to varicella.The cost for screening and then vaccination was €9942.5 for MMR and €6815 for varicella.The cost for vaccination without screening was €2422.5 for MMR and €15900 for varicella.The cost of vaccination without screening was significantly expensive (cost difference: €9085) for varicella, however cheap for MMR (cost difference: €7520).

## Conclusion

For the immunization against MMRV in HCWs, immunity rate, screening and vaccine cost and side effects should be considered in developing countries. Cheaper vaccines may be cost effective for vaccination without screening, however prescreening may be rule out side effects.

## Disclosure of interest

None declared.

